# Ruptured splenic artery aneurysm detected by emergency ultrasound—a case report

**DOI:** 10.1186/s13089-015-0026-4

**Published:** 2015-06-10

**Authors:** W L Lo, K L Mok

**Affiliations:** Accident and Emergency Department, Ruttonjee Hospital, Wanchai, Hong Kong

**Keywords:** Splenic artery, Aneurysm, Pseudoaneurysm, Rupture, Ultrasound, ED

## Abstract

Splenic artery aneurysm is a rare but a potentially fatal condition. It is usually asymptomatic until it ruptures. Here, we present a case of ruptured splenic artery aneurysm in a 59-year-old gentleman presenting with epigastric pain and hypovolemic shock. The diagnosis was made by emergency ultrasound and CT scan, and he was managed by laparotomy and excision of the splenic artery aneurysm. Priorities in patient management lie in rapid resuscitation, diagnostic imaging, surgical consultation, and subsequent laparotomy. Pitfalls should be borne in mind to differentiate splenic artery aneurysm from abdominal aortic aneurysm when using the emergency ultrasound.

## Background

Splenic artery aneurysm (SAA) is an uncommon, but potentially life-threatening, condition. It occurs in approximately 1 % of the population [1]. SAA accounts for approximately 60 % of all visceral arterial aneurysms [2]. It is the third most common intra-abdominal aneurysm, following aortic and iliac arteries aneurysms [3]. Clinical presentation of a SAA is variable, with most patients being asymptomatic and detected incidentally [4]. If it ruptures, though rare, it may manifest as hypovolemic shock and has a very high mortality rate (25–70 %) [5]. Splenic artery pseudoaneurysm is less prevalent than true SAA [3]. It differs from true SAA in that the dilatation occurs following the disruption of one or more layers of the vessel wall and is usually secondary to a local inflammatory process such as pancreatitis [6].

We describe a case of ruptured splenic artery pseudoaneurysm who presented to our department with epigastric pain and hypovolemic shock.

## Case presentation

A 59-year-old gentleman presented to ED with sudden onset of epigastric pain and back pain, accompanied by dizziness and sweating. He had known history of hypertension and open appendicectomy more than 20 years ago. He was pale and in shock at the triage, with blood pressure 55/34 mmHg and pulse 105 bpm. Abdominal examination revealed distended abdomen with tenderness over the epigastrium. The rectum was empty on per rectal examination.

Electrocardiogram showed a sinus rhythm with no acute ischemic change. Chest X-ray showed clear lung fields and no free gas under diaphragm. KUB was unremarkable. Fluid challenge was done, but the patient was still in shock.

**Fig. 1:**
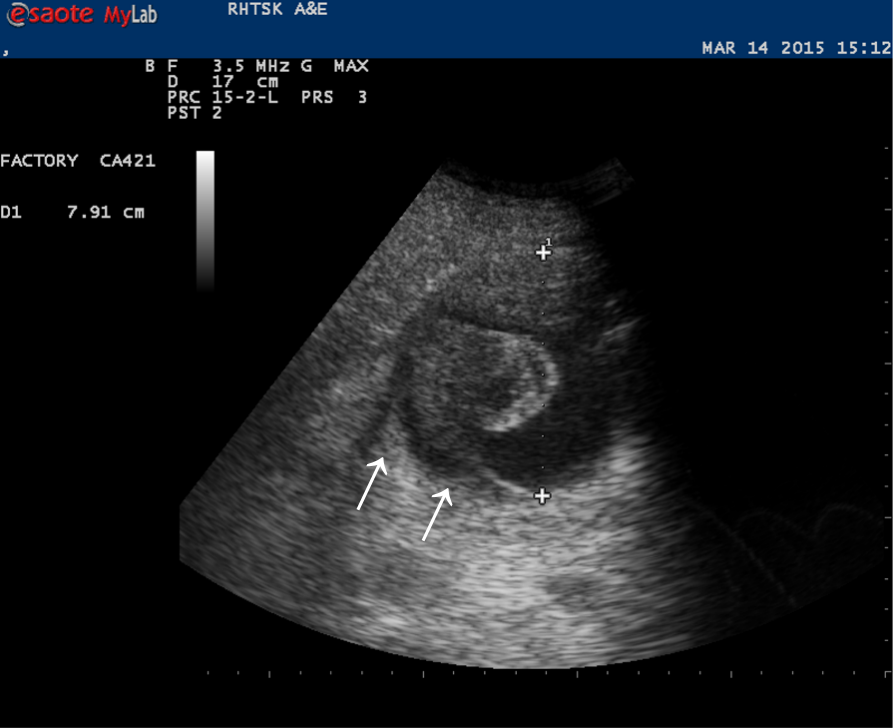
Sonography image showing a 7.9-cm mass with free fluid [←] around it

**Fig. 2:**
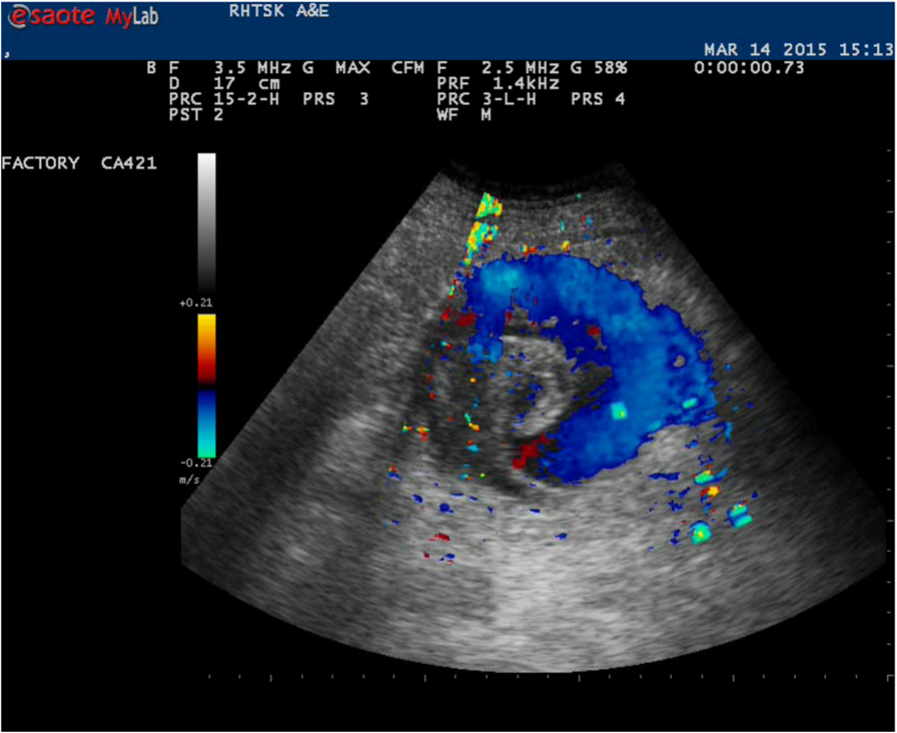
Strong Doppler signal was detected inside the lesion, signifying strong blood flow inside

Bedside ultrasound of the abdomen showed a 7.9-cm mass with strong Doppler flow signals inside. Free fluid was noted in the Morrison pouch and around the mass (Figs. 1 and 2). The mass was found to be discrete—there was no connection to the aorta when chasing caudally with the ultrasound.

**Fig. 3:**
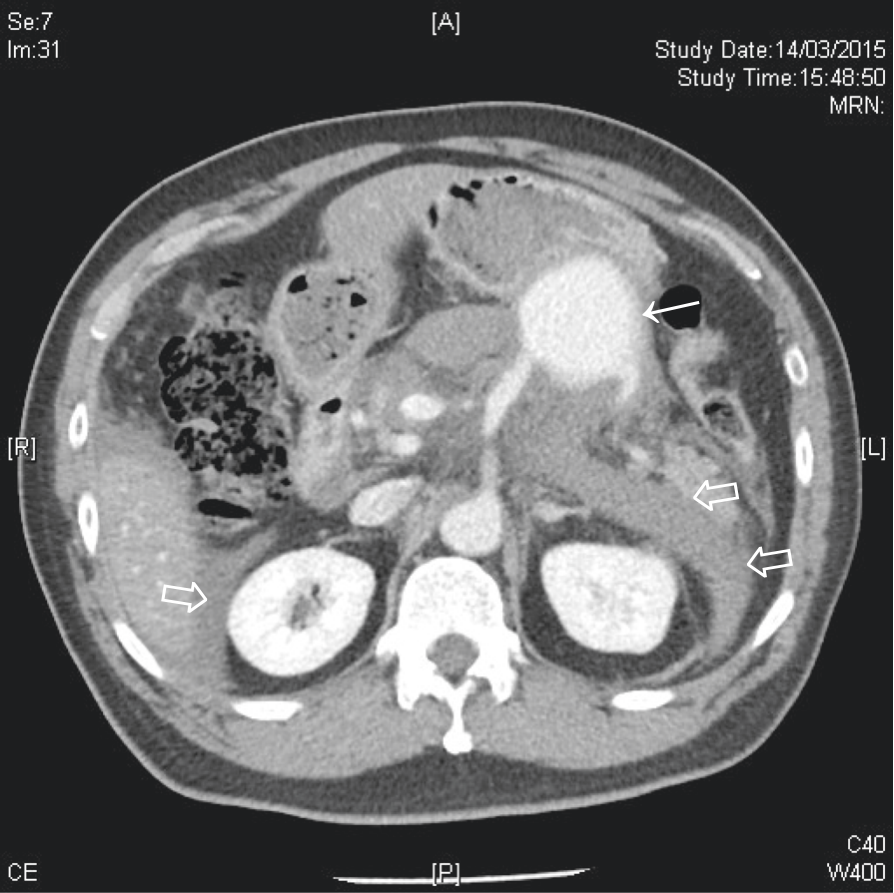
CT scan of the abdomen with IV contrast showing a contrast-enhanced mass [←] at the splenic artery (branching from the superior mesenteric artery) compatible with a splenic artery pseudoaneurysm. Hemoretroperitoneum and hemoperitoneum were demonstrated [⇦]

**Fig. 4:**
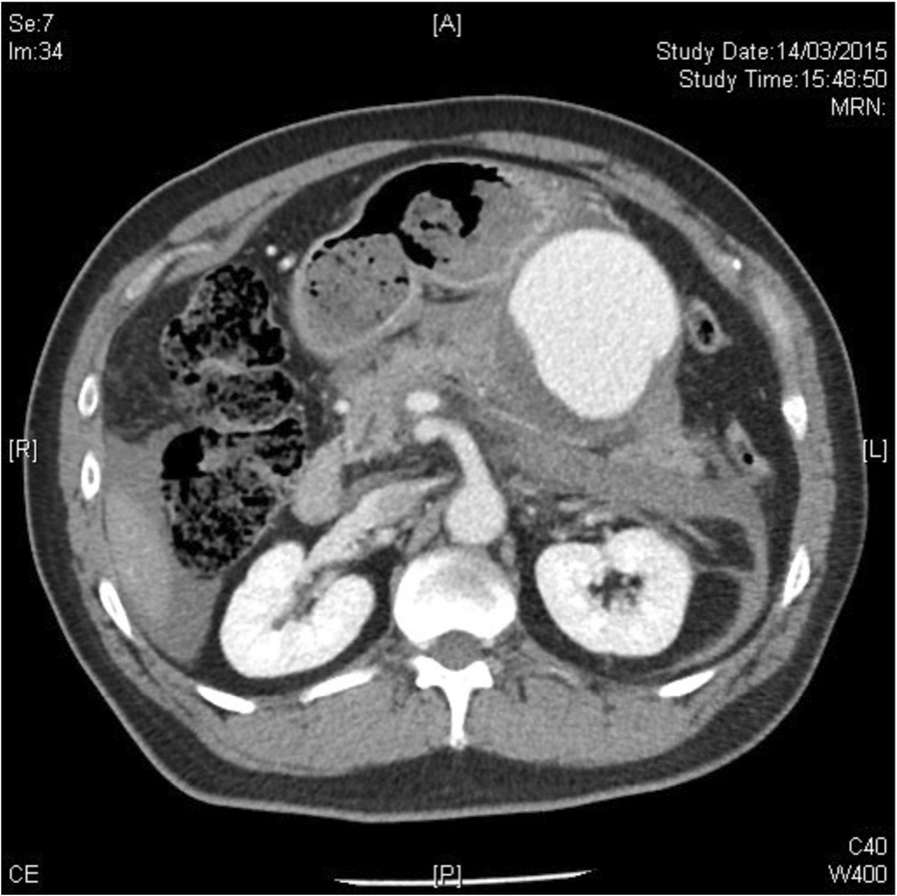
CT scan of the abdomen with IV contrast showing the splenic artery pseudoaneurysm in close proximity to the pancreas, with a partial thrombus and hyperdense hematoma. Hemoretroperitoneum and hemoperitoneum were again demonstrated

An on-call surgeon was urgently consulted, and ruptured abdominal aortic aneurysm was suspected. Urgent CT scan of the abdomen with IV contrast showed a large splenic artery pseudoaneurysm measuring up to 8.6 cm × 8.0 cm × 8.7 cm (AP × TS × LS) located within a pancreatic pseudocyst. Hemoretroperitoneum and hemoperitoneum was demonstrated. There was no active contrast extravasation (Figs. 3 and 4).

The patient was subsequently taken to an operation theater for emergency laparotomy and exploration. Three hundred milliliters hemoperitoneum and an 8-cm aneurysm being eroded by a pancreatic pseudocyst in the mid-segment of the splenic artery were found intraoperatively. Hemostasis and excision of the splenic artery aneurysm was successfully performed. The patient was then transferred to the ICU for close observation. He had an uneventful postoperative course and was discharged home on the seventh postoperative day.

## Discussion

### Splenic artery aneurysm

Splenic artery aneurysm (SAA) is defined as an abnormal dilatation of the splenic artery more than 1 cm in diameter. It was first described on cadavers in 1770 by Beaussier [7]. It accounts for approximately 60 % of all visceral arterial aneurysms [2]. It is the third most common intra-abdominal aneurysm, following aortic and iliac artery aneurysms [3]. SAA is rarely seen with a prevalence of 1 % [1]. It is four times more common in females compared to males [8–10]. Risk factors correlating to the development of SAA include fibromuscular dysplasia, collagen vascular diseases, female gender, history of multiple pregnancies, and portal hypertension, although the pathogenesis is not fully understood [11].

Splenic artery pseudoaneurysms are less prevalent than true SAA. They differ from true SAA in that the dilatation occurs following the disruption of one or more layers of the vessel wall. Splenic artery accounts for the majority of splanchnic pseudoaneurysms. Unlike true SAA, they have a slight male predominance. The underlying causes in most of the cases are trauma, infection, or weakening of the splenic artery wall from exposure to pancreatic enzymes. The latter is usually associated with pancreatic anastomotic leaks, severe pancreatitis, and pancreatic pseudocysts [3]. However, in our patient, no risk factors could be identified. He did not have previous history suggestive of pancreatitis but presented as acute rupture of splenic artery pseudoaneurysm. A pancreatic pseudocyst was found intraoperatively. It can be postulated that he might have subclinical pancreatitis in the past resulting in pseudocyst formation. The pseudocyst caused erosion into the splenic artery, leading to formation of a pseudoaneurysm.

Patients with SAA are usually asymptomatic, only 20 % of then have symptoms such as abdominal pain, chest pain, and most are diagnosed incidentally. SAA can be complicated by rupture resulting in hypovolemic shock as illustrated in your case. It can be fatal if not treated properly and timely. It can rupture freely in the peritoneal cavity, in the gastrointestinal (GI) tract causing GI hemorrhage or eroding into surrounding structures, such as the splenic vein, resulting in a splenic arteriovenous fistula [12]. Double rupture phenomenon may occur, in which the aneurysm first ruptures into the lesser sac with mild clinical symptoms then the blood overflows into the peritoneal cavity through the Winslow foramen with hemorrhagic shock [5, 13].

The significance of diagnosing and treating SAA lies in the potential risk for rupture and life-threatening hemorrhage, which occur in 10 % of cases with a mortality rate of 10–25 % in nonpregnant patients and up to 70 % during pregnancy [14]. The risk of rupture however is much higher for aneurysms larger than 2 cm in diameter [15].

### ED interventions for symptomatic splenic artery aneurysms

The emergency physician’s role in the care of a patient with an acute rupture of a splenic artery aneurysm lies largely in making the diagnosis and urgent surgical consultation. Standard resuscitative maneuvers (insertion of two large-bore IV catheters, initiation of cardiac monitoring, and administration of supplemental oxygen) are required. Fluid resuscitation is needed if the patient is hemodynamically unstable. However, caution should be taken to avoid over-resuscitation which will potentially cause more bleeding if bleeding is still not under control.

Imaging modalities for diagnosing splenic artery aneurysm include ultrasound, pulsed Doppler, CT, MRI, and abdominal aortic arteriography, which is the gold standard [16]. In our case, bedside ultrasound findings suggested rupture of the aneurysm, and subsequent CT scan confirmed the diagnosis of rupture of splenic artery pseudoaneurysm.

Rapid bedside ultrasound is useful to demonstrate the aneurysm and associated free fluid inside the abdomen. It is ideal for patients in an unstable condition who cannot undergo CT scanning. Emergency ultrasound is noninvasive, can be rapidly deployed, and does not entail removal of the patient from the resuscitation area [17]. Ultrasound is also radiation-free, thus particularly useful in pregnancy. However, it is operator independent and its sensitivity is significantly degraded in the presence of obesity, gaseous distension, arteriosclerosis, and small aneurysms [3].

CT scanning with IV contrast material is useful to demonstrate the anatomic details of the aneurysm in three-dimensions, associated retroperitoneal hemorrhage, and associated underlying diseases. CT scanning should be obtained in patients in stable condition.

**Fig. 5:**
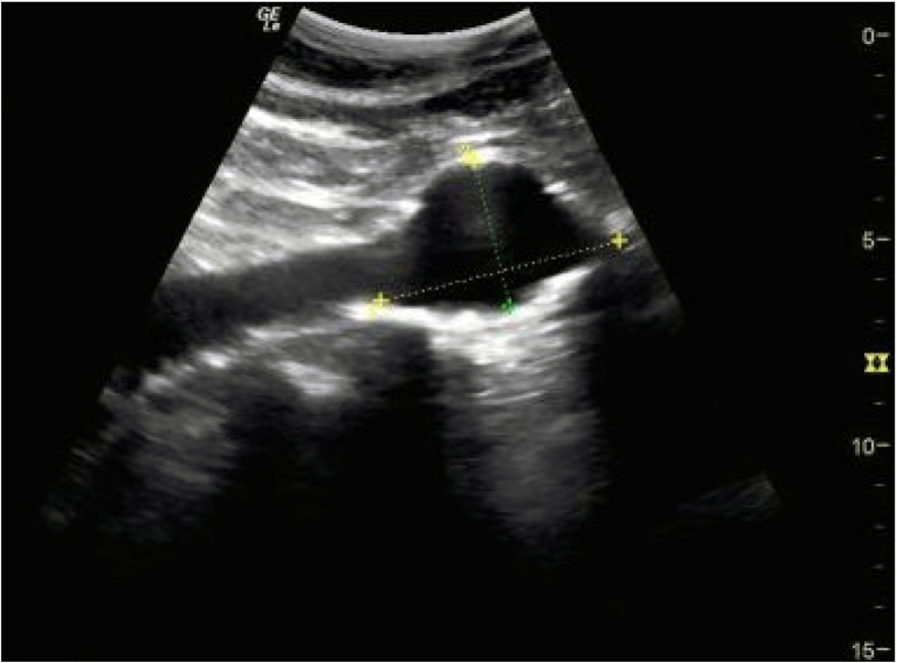
Sonography image showing an AAA in a longitudinal section. It shows the continuity of the aneurysm with the rest of the abdominal aorta

The management of ruptured SAA is similar to that of ruptured abdominal artery aneurysm (AAA). Emergency physicians are familiar with using ultrasound to diagnose AAA. With the wide availability of ultrasound in EDs nowadays, SAA can be readily picked up under appropriate clinical settings. However, pitfalls should be borne in mind to differentiate SAA from AAA (Fig. 5). In our case, color Doppler sonography showed a large mass with strong Doppler flow inside, suggestive of an aneurysm. To differentiate a visceral artery aneurysm from an AAA, the aneurysm should be discrete, and no continuity should be demonstrated when chasing the entire abdominal aorta down to bifurcation into both common iliac arteries.

### Update management of SAA

SAA with features suggestive of low risk for rupture may be successfully managed without intervention. Radiological follow-up with six monthly ultrasound or CT scans should be mandatory to assess progression of the aneurysm. Active intervention should be considered if the aneurysm is symptomatic, enlarging, more than 2 cm in diameter or if found in pregnancy or childbearing age. All false aneurysms of the splenic artery should be treated as soon as possible, irrespective of size, symptoms, or rupture [3].

The therapeutic options are either surgical or endovascular intervention.

Endovascular procedure, which includes embolization or stent graft application, is considered as a first choice of treatment for splenic artery aneurysm [18]. The choice between embolization and stent grafting should be dependent on the shape, size, and site of the SAA as well as the local expertise [3].

Surgical intervention is considered the conventional option of treatment in most centers especially in case of rupture [14]. The options include excision, ligation, or revascularization, with or without splenectomy [12]. Laparoscopic approach may be considered if radiation exposure is contraindicated, such as pregnancy, or where endovascular techniques either fail or are not available [3].

## Conclusion

SAA is uncommonly presented to the emergency department, but the outcome can be catastrophic if it ruptures. Rapid resuscitation, diagnostic imaging, surgical consultation, and subsequent laparotomy remain the priorities in patient management. Management of rupture SAA is similar to that of rupture AAA. However, pitfalls should be borne in mind to differentiate SAA from AAA when using the emergency ultrasound.

## Consent

Written informed consent was obtained from the patient for publication of this case report and any accompanying images. A copy of the written consent is available for review by the Editor-in-Chief of this journal.
